# Transkranielle elektrische Hirnstimulationsverfahren zur Behandlung der Negativsymptomatik bei Schizophrenie

**DOI:** 10.1007/s00115-021-01065-5

**Published:** 2021-01-25

**Authors:** Nikolas Haller, Alkomiet Hasan, Frank Padberg, Wolfgang Strube, Leandro da Costa Lane Valiengo, Andre R. Brunoni, Jerome Brunelin, Ulrich Palm

**Affiliations:** 1 Klinik für Psychiatrie und Psychotherapie, Klinikum der Universität München, München, Deutschland; 2 Klinik für Psychiatrie, Psychotherapie und Psychosomatik, Universität Augsburg, Medizinische Fakultät, BKH Augsburg, Augsburg, Deutschland; 3 Laboratory of Neurosciences (LIM-27), Department and Institute of Psychiatry, Faculdade de Medicina, Universidade de São Paulo, São Paulo, Brasilien; 4 CH le Vinatier, INSERM U 1028, CNRS UMR 5292, PSYR2 Team, Centre de recherche en neuroscience de Lyon, Université de Lyon, Lyon, Frankreich; 5 Medical Park Chiemseeblick, Rasthausstr. 25, 83233 Bernau-Felden, Deutschland

**Keywords:** Nichtinvasive Hirnstimulation, tDCS, tACS, tRNS, Kognition, Noninvasive brain stimulation, tDCS, tACS, tRNS, Cognition

## Abstract

Über die letzten Jahre entwickelten sich Neuromodulationsverfahren zu einer dritten Säule neben Pharmakotherapie und Psychotherapie in der Behandlung psychischer Erkrankungen. Besonders in der Behandlung von Menschen mit einer Schizophrenie könnten Hirnstimulationsverfahren eine Alternative oder Ergänzung zu den etablierten Therapiestrategien darstellen. Die meist vorhandenen Positivsymptome können zumeist mit Antipsychotika adäquat behandelt werden. Gerade bei Patienten mit Schizophrenie besitzen jedoch Negativsymptome einen überdauernden Krankheitswert und beeinflussen den Verlauf durch globale Antriebsverarmung und beeinträchtigte Kognition im alltäglichen Leben negativ. Dieser Übersichtsartikel stellt eine Zusammenfassung über die verschiedenen nichtinvasiven Hirnstimulationsverfahren transkranielle Gleichstromstimulation (transcranial direct current stimulation, tDCS), Wechselstromstimulation (transcranial alternating current stimulation, tACS) sowie Rauschstromstimulation (transcranial random noise stimulation, tRNS) zur Behandlung der Negativsymptomatik bei Schizophrenie dar. Die neuen transkraniellen Hirnstimulationsverfahren könnten dabei helfen, gestörte neuronale Vernetzungen wieder herzustellen und die Konnektivität vor allem der dorsolateralen präfrontalen Anteile des Kortex zu verbessern. Einige Studien weisen auf eine Verbesserung der Negativsymptome durch Behandlung mit tDCS, tACS bzw. tRNS hin und könnten so neue Therapiemöglichkeiten in der Behandlung der Schizophrenie darstellen.

## Abstract (en)

In recent years noninvasive brain stimulation (NIBS) applications have emerged as a third and novel treatment option alongside psychopharmacology and psychotherapy in the treatment of mental diseases. It is assumed that NIBS could represent a supplement or (in some indications) even replacement to established therapeutic strategies, e.g. in disorders with high resistance to current treatment regimens, such as negative symptoms or cognitive impairments in schizophrenia. Although positive symptoms in schizophrenia can be treated sufficiently with antipsychotic drugs, patients with negative symptoms frequently suffer from persistent lack of impetus, cognitive decline, social withdrawal and loss of global functioning in the activities of daily life; however, in these cases, current treatment strategies exert only moderate effects, and new treatment options are urgently needed. This review article provides a summary of the clinical effects of new electrical NIBS methods, e.g. transcranial direct current stimulation (tDCS), transcranial alternating current stimulation (tACS), and transcranial random noise stimulation (tRNS) for the treatment of negative symptoms in schizophrenia. These new NIBS methods could help restore the disrupted neuronal networks and improve disturbed connectivity, especially of the left dorsolateral prefrontal cortex and left temporoparietal junction. Promising results are reported for the treatment of negative symptoms with tDCS, tACS and tRNS and could thus represent new therapeutic options in the treatment of schizophrenia.

## Hintergrund

Weltweit sind ca. 1–2 % der Bevölkerung an Schizophrenie erkrankt [63]. Diese schwere psychiatrische Erkrankung, bei der die Betroffenen unter anderem an ausgeprägten kognitiven Einschränkungen, Antriebsstörung, Affektstörung und sozialer Vereinsamung leiden, geht mit einer stark erhöhten Mortalität einher [27]. Die Lebenszeit der Menschen mit einer Schizophrenie ist im Vergleich zur Normalbevölkerung um ca. 10 Jahre verringert, wobei die Hauptgründe hierfür in der erhöhten Suizidalität [58] und den nicht oder unzureichend behandelten körperlichen Begleiterkrankungen liegen [41]. Da die Schizophrenie eine meist chronisch verlaufende und schwer zu behandelnde, komplexe Erkrankung ist, stellt sie zudem eine große sozioökonomische Herausforderung dar [39]. Neben den meist klassischerweise und zur Diagnosestellung hauptsächlich herangezogenen Positivsymptomen [44] bestehen bei knapp der Hälfte der erwachsenen Patienten Negativsymptome [62]. Diese äußern sich vor allem in Affektarmut, einer Sprachverarmung, kognitiven und depressiven Symptomen [4] und sind als überdauernde und schwer zu therapierende Symptome der wesentliche Grund für die Bürde der Erkrankung und die mit ihr verbundene soziale Isolation und Arbeitsunfähigkeit [11].

In der pharmakologischen Behandlung der Positiv- wie Negativsymptome der Schizophrenie zeichneten sich seit Jahren einige Fortschritte ab [61] und auch Cariprazin [49] schien einen Durchbruch zu versprechen, jedoch wurde dem Medikament vom Gemeinsamen Bundesausschuss (GBA) nur ein geringer Zusatznutzen bei der Behandlung der Negativsymptomatik zuerkannt. Insgesamt ist die Effektivität der Antipsychotika unverändert, bei jedoch verbesserter Verträglichkeit. Weiterhin kommt es jedoch bei rund einem Drittel der Patienten nur zu einer unvollständigen Remission [48]. Dementsprechend ist der Leidensdruck der Betroffenen weiterhin hoch und der Fokus der Forschung liegt auf den oft therapieresistenten Negativsymptomen. Gerade im langfristigen Disease-Management haben die Negativsymptome einen besonderen Stellenwert erlangt [22, 23], da aus Studien gut belegt ist, dass Positivsymptome über den Zeitraum der Erkrankung eher abnehmen [25], Negativsymptome hingegen oft für einen chronischen Verlauf der mit einer Schizophrenie verbundenen Einschränkungen für die Betroffenen verantwortlich sind [9].

Wichtig hierbei ist, dass sich vor allem medikamentös schlecht beherrschbare kognitive Dysfunktionen auf die Gesamtsymptomatik der betroffenen Patienten auswirken [12]. Bei zwei Dritteln der Patienten findet sich außerdem eine globale Kognitionsminderung, die zusätzlich einen sozialen Rückzug bedingt [38]. Diese Vermischung von kognitiven und Negativsymptomen ist klinisch nicht leicht zu unterscheiden. Wesentliche Ursachen für die fehlende Trennschärfe zwischen kognitiven und Negativsymptomen ist die unklare Ätiologie (gemeinsam oder getrennt) sowie die wechselseitige Überlappung und Bedingung, wobei die kognitiven Defizite eher mit dem desorganisierten Typus als mit den Negativsymptomen zusammenhängen [21].

In der aktualisierten S3-Leitlinie Schizophrenie [22] stellen die pharmakologische und psychotherapeutische Therapie zwar zentrale Stützpfeiler der Therapie der Schizophrenie dar, insbesondere in der Therapie der Negativsymptomatik stehen mit kognitiver Verhaltenstherapie bzw. Training sozialer Kompetenzen zwei Interventionen mit einem Empfehlungsgrad A zur Verfügung, während die Gabe von Antidepressiva lediglich einen Empfehlungsgrad B erreicht. Gerade bei therapieresistenten Verläufen sollten jedoch weitere Behandlungsstrategien erwogen bzw. entwickelt werden [5]. Hierbei werden bereits seit einigen Jahren nichtinvasive Hirnstimulationsverfahren (NIBS, „non-invasive brain stimulation“) beforscht. Neben der minimal-invasiven und fest etablierten Elektrokonvulsionstherapie hat die seit nunmehr über 30 Jahren beforschte repetitive transkranielle Magnetstimulation (rTMS) einen gewissen Stellenwert erhalten [24, 37], wobei eine neuere Metaanalyse von Aleman et al. [3] eine Wirksamkeit aktiver rTMS gegenüber Placebo nachweisen konnte. Zu diesen beiden Verfahren liegen bereits neuere Übersichtsarbeiten vor [24, 37], sodass auf Elektrokonvulsionstherapie und Magnetstimulation hier nicht eingegangen wird.

Neuere, auf verschiedenen Stromarten beruhende Verfahren wie tDCS („transcranial direct current stimulation“, transkranielle Gleichstromstimulation), tACS („transcranial alternating current stimulation“, transkranielle Wechselstromstimulation) oder tRNS („transcranial random noise stimulation“, transkranielle Rauschstromstimulation) haben in den letzten Jahren vielversprechende Ergebnisse erbracht, wobei viele Pilotergebnisse erst durch große kontrollierte Studien bestätigt werden müssen. Die neurobiologische Hypothese einer elektrischen Stimulation meist präfrontaler und parietaler Kortexbereiche ist die Annahme einer frontothalamoparietalen Dysfunktion, die gleichsam für die Negativsymptomatik wie auch für die Kognitionseinschränkungen als verantwortlich angesehen wird [6, 59]. Eine Dysfunktion kortikaler Bereiche mit linksseitiger temporoparietaler Überaktivierung vor allem in Verbindung mit akustischen Halluzinationen ist bereits seit vielen Jahren bekannt [31]. Weiterhin gibt es Hinweise, dass bei Negativsymptomatik ein Funktionsverlust des vor allem linken dorsolateralen präfrontalen Kortex (DLPFC) besteht [59]. Diese Veränderung führt zu einer gestörten Interaktion beider Hirnareale, zu einer frontotemporalen Dyskonnektivität [41, 60]. An dieser Stelle setzen neuromodulatorische Hirnstimulationsverfahren an, die durch eine Änderung neuronaler Aktivität, zunächst in einem lokalen Netzwerk und im Weiteren durch Veränderung der Interkonnektivität entfernterer Hirnareale, eine Veränderung der langfristigen Neuroplastizität erreichen wollen.

**Figure 1:**
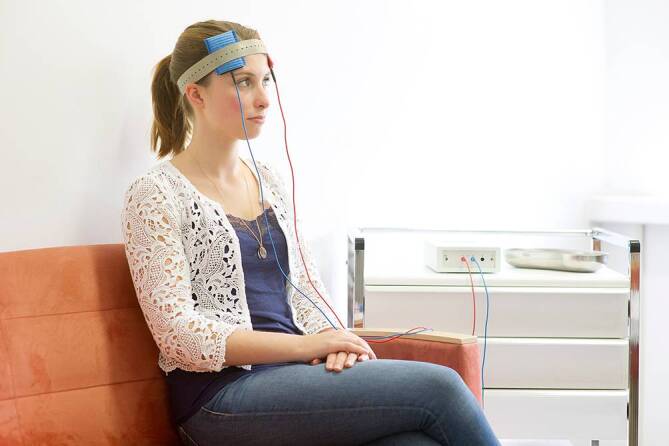

In Abb. 1 wird eine typische Montage zweier Platten-Schwammelektroden (5 × 7 = 35 cm^2^) an beiden dorsolateralen präfrontalen Kortizes gezeigt, wobei bei der tDCS die rote Elektrode für die linksseitig montierte Anode steht, die blaue Elektrode für die rechtsseitig montierte Kathode (häufig auch Referenzelektrode genannt).

Die technischen Spezifikationen der drei erwähnten elektrischen Stimulationsarten unterscheiden sich erheblich, weshalb auch differenzielle Wirkungen zu erwarten sind [40]. In Tab. 1 werden die Unterschiede und Gemeinsamkeiten kurz zusammengefasst.

**Table 1:** 

	tDCS	tACS	tRNS
Stromart	Gleichstrom	Wechselstrom	Rauschstrom
Frequenz	Statisch	Frei wählbar	0–640 Hz
Stromstärke	1–2 mA	1–2 mA	1–2 mA
Charakteristik	Statisches Stromfeld mit negativer oder positiver Polarität	Sinusoidaler Phasenwechsel	Zufallsgesteuerte rasche Amplitudenwechsel
Anwendungsdauer	20–30 min	10–20 min	Unklar (10–20 min?)

## Methodik

**Figure 2:**
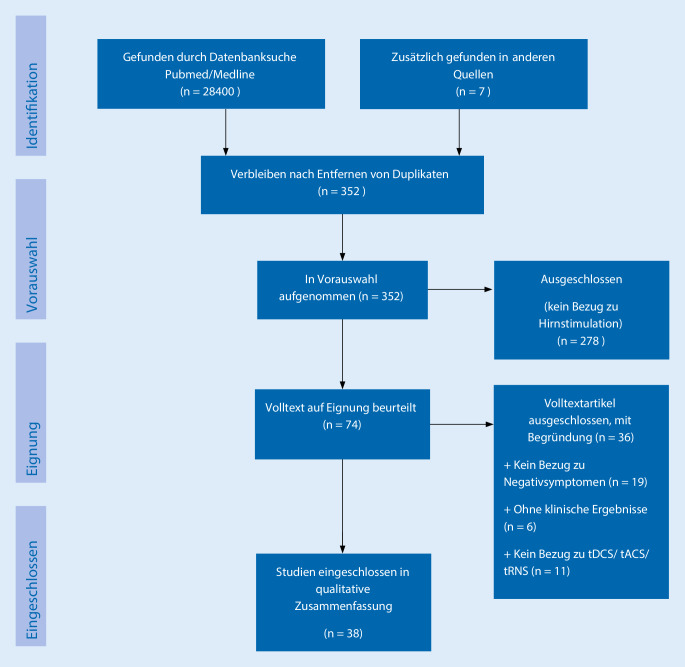

Die Datenbanken PubMed/Medline wurden mit den Suchbegriffen „brain stimulation“, „non-invasive“, „schizophrenia“, „negative symptoms“ jeweils als Kreuzkombinationen durchsucht. Gesucht wurde für den Zeitraum 01.01.2000 bis 30.08.2020 und nur für englischsprachige Literatur. Die gefundene Literatur wurde auf einen Bezug zu tDCS, tACS und tRNS geprüft. Nur kontrollierte klinische („randomized controlled clinical trial“) oder offene Studien („open label studies“), Fallserien („case series“), Einzelfallberichte („single case report“) und Übersichtsarbeiten/Metaanalysen („review article“/„meta-analysis“) wurden berücksichtigt, Kongressbeiträge („congress proceeding“) sowie publizierte Studienprotokolle („trial protocol“) wurden ausgeschlossen. Die Literaturverzeichnisse der ausgewählten Studien wurden nach weiterer relevanter Literatur durchsucht. Insgesamt konnten aus über 28.000 gefundenen Datenbankeinträgen 38 Studien identifiziert werden, die in die qualitative Synthese Eingang fanden (Abb. 2).

## tDCS

Als erstes neues Hirnstimulationsverfahren wurde die tDCS bei Positivsymptomen (akustische Halluzinationen) eingesetzt, mit dem Ziel die oben beschriebene Dysfunktion im Sinne einer linksseitigen temporoparietalen Überaktivierung zu verbessern. Als Nebeneffekt konnte in einigen Studien eine Verbesserung von Negativsymptomen beobachtet werden. Die erste randomisierte klinische Studie wurde 2012 von Brunelin et al. publiziert und zielte auf eine Verbesserung therapieresistenter akustischer Halluzinationen ab [8]. Hierzu wurden 30 Patienten monohemisphärisch kathodal über dem linken temporoparietalen Kortex und anodal über dem linken DLPFC über 5 Tage 2‑mal täglich 20 min lang mit tDCS (2 mA) oder Placebostimulation behandelt. Die Patienten der Verumgruppe zeigten bis zu drei Monate nach der Stimulationsserie eine signifikante Reduktion akustischer Halluzinationen im Vergleich zu Placebo. Zusätzlich zeigten die Patienten der Verumgruppe eine Verringerung der Negativsymptomatik. Mondino et al. [46] zeigte mit der gleichen Verfahrensweise ähnliche Ergebnisse. Dies stellte sich auch in den Subskalen des PANSS (Positive and Negative Symptom Scale) dar, welche eine Verringerung der Negativsymptome um 14,4 % nach tDCS aufwiesen, jedoch keine Reduktion nach Placebostimulation.

Fitzgerald et al. [13] beschrieb im Gegensatz hierzu in einer Studie mit 24 Patienten mit behandlungsresistenten Negativsymptomen und persistierenden akustischen Halluzinationen keine Überlegenheit der tDCS gegenüber einer ebenso durchgeführten Scheinstimulation (Placebokondition in Stimulationsstudien, „sham stimulation“).

Eine Abnahme akustischer Halluzinationen bei 26 Patienten mit Schizophrenie und schizoaffektiver Störung beschrieben Fröhlich et al. [14] im Rahmen einer randomisierten, placebokontrollierten Studie mit der Anode über dem linken DLPFC (2 mA, 20 min, 5 Sitzungen) und der Kathode über dem linken temporoparietalen Übergang. Sowohl in der Gruppe der mit Verum-tDCS stimulierten als auch in der Gruppe der Patienten mit Scheinstimulation kam es zu einer signifikanten Abnahme akustischer Halluzinationen, was sich anhand der Ergebnisse des Auditory Hallucination Rating Scale (AHRS) darstellen ließ. Der PANSS und darunter auch die Negativsymptome zeigten keinen signifikanten Unterschied zwischen den randomisierten Gruppen.

Chang et al. [10] stimulierten 60 Patienten mit medikamentenresistenter Schizophrenie und akustischen Halluzinationen 2‑mal täglich mit tDCS (2 mA) an 5 aufeinanderfolgenden Tagen mit anodaler Stimulation über F3 und kathodaler Stimulation über TP3. Es konnte hierbei keine Verbesserung der akustischen Halluzinationen im AHRS sowie keine Symptomverbesserung der Schizophrenie im PANSS erreicht werden, allerdings verbesserte sich die Krankheitseinsicht gemessen am SUMD (Scale to Assess Unawareness in Mental Disorder in Schizophrenia).

In einer frühen Übersichtsarbeit wurde auf die Wirksamkeit einer inhibierenden Stimulation des auditorischen Kortex zur Verringerung akustischer Halluzinationen und einer exzitatorischen Stimulation des DLPFC zur Verbesserung der Negativsymptomatik hingewiesen, wobei in den frühen Studien jeweils der PANSS als Messinstrument für die gesamte Krankheitsschwere und zur Abbildung überwiegender Positivsymptomatik in den untersuchten Stichproben verwendet wurde [45].

In einer ersten, auf die Negativsymptome fokussierten Studie verwendeten Gomes et al. den SANS (Scale for the Assessment of Negative Symptoms) als spezifischen Fragebogen und konnten mittels bihemisphärischer tDCS, bei der die Anode über dem linken DLPFC und die Kathode kontralateral angebracht wurde, eine Verbesserung der Negativsymptome beobachten [17]. Der CDSS (Calgary Depression Scale in Schizophrenia), als Bewertungsinstrument der Depression bei Patienten mit Schizophrenie, änderte sich jedoch in der Verumgruppe nicht. Insgesamt war die Anzahl von 15 in die Studie eingeschlossenen Patienten gering, sodass von ihr zunächst Pilotcharakter ausging.

Palm et al. [54] führten eine randomisierte, kontrollierte Studie an 20 Patienten durch, wobei diese entweder tDCS oder Scheinstimulation mit der Anode über dem linken DLPFC und der Kathode kontralateral erhielten. Nach 10 Behandlungen mit tDCS (2 mA, 20 min) zeigte sich eine signifikante Verringerung der Negativsymptome der SANS und der PANSS Skalen.

Jeon et al. [32] konnten eine Verbesserung des Arbeitsgedächtnisses und depressiver Symptomatik, hingegen keine Veränderung der Negativsymptome bei 56 Schizophreniepatienten nachweisen. Hier wurden die Patienten mit tDCS (2 mA, Anode F3, Kathode F4) an 10 aufeinanderfolgenden Wochentagen einmal täglich 30 min stimuliert.

In einer Studie von Lindenmayer et al. [42] wurden 28 Patienten in 20 tDCS-Sitzungen (Kathode über TP3 und Anode zwischen F3 und FP1) mit behandlungsresistenter Schizophrenie untersucht. Hierbei zeigte sich eine signifikante Verringerung der akustischen Halluzinationen, wohingegen PANSS und die Negativsymptomatik der Patienten unverändert blieben.

Studien mit der bisher größten Anzahl an Patienten (89 bzw. 100) führten Valiengo et al. und Kantrowitz et al. durch.

**Figure 3:**
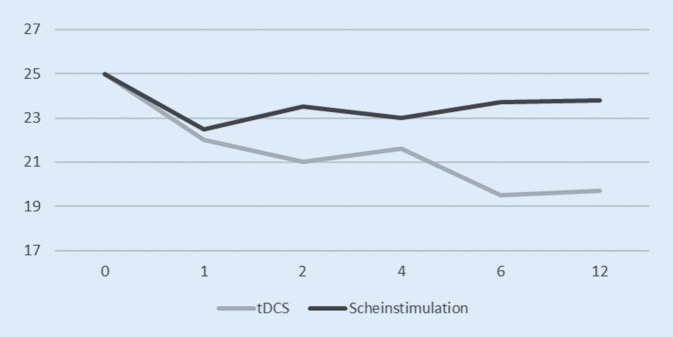

Hierbei wurde eine tDCS (2 mA) über F3 (Anode) und TP3 (Kathode) appliziert. Insgesamt wurden 10 Behandlungen an 5 aufeinanderfolgenden Tagen 2‑mal täglich angewendet. Rationale dieser Elektrodenplatzierung ist der Nachweis einer gestörten funktionellen Interkonnektivität zwischen linkem DLPFC und auditorischem Kortex in einer früheren Studie von Mondino et al. [46], mit dem Ziel einer Wiederherstellung der Balance zwischen beiden Hirnarealen, indem eine eventuell vorhandene halluzinatorische Restkomponente vermindert wird. Kantrowitz et al. [35] berichteten hierbei von einer Verringerung akustischer Halluzinationen jedoch ohne Abnahme negativer Symptome. Valiengo et al. [71] berichteten im Gegensatz hierzu von einer Verminderung negativer Symptome, die sich in der Negativsubskala der PANSS abzeichnete (Abb. 3).

Eine Erklärung für die Dissonanz dieser Ergebnisse findet sich wahrscheinlich im jeweiligen Studiendesign, wobei sich im rekrutierten Patientenkollektiv von Valiengo et al. bei nur rund 36 % der Patienten Halluzinationen zeigten. Andererseits untersuchten Kantrowitz et al. eine Stichprobe von Patienten, welche eine geringere Negativsymptomatik aufwiesen.

Kim et al. [36] führten 2019 eine Metaanalyse über die Auswirkungen der tDCS bei Patienten mit Schizophrenie durch. Hinsichtlich der Negativsymptomatik zeigten 9 Studien mit 313 Patienten, dass die Wirksamkeit der Stimulation erst ab einer Mindestanzahl von 10 Behandlungen eine Verbesserung mit sich bringt.

Insgesamt wurden zur Prüfung der Wirksamkeit der tDCS auf Negativsymptomatik bei Schizophrenie in den letzten Jahren viele Einzelfallberichte sowie offene Studien und randomisierte, kontrollierte klinische Studien durchgeführt. Tab. 2 zeigt eine Übersicht über die bisher durchgeführten randomisierten, kontrollierten klinischen Studien. Offene Studien und Fallberichte zur tDCS bei Negativsymptomatik werden aufgrund der Fülle hier nicht gesondert berichtet, hierzu wird auf einen englischen Übersichtsartikel verwiesen [45].

**Table 2:** 

Autor	Patientenanzahl	Anode	Kathode	Stromstärke (mA)/Elektrodengröße (cm^2^)	Anzahl der Stimulationen	Ergebnisse
Brunelin et al. 2012 [8]	30	F3	TP3	2/35	10	Positiv^a^
Fitzgerald et al. 2014 [13]	24	F3 (F4)	TP3 (TP4)	2/35	15	Negativ^a^
Gomes et al. 2015 [17]	15	F3	F4	2/35	10	Positiv
Mondino et al. 2015 [46]	28^a^	F3	TP3	2/35	10	Positiv^a^
Fröhlich et al. 2016 [14]	26	F3	TP3	2/35	5	Negativ^a^
Palm et al. 2016 [55]	20	F3	FP2	2/35	10	Positiv
Jeon et al. 2018 [32]	56	F3	F4	2/35	10	Negativ
Chang et al. 2018 [10]	60	F3	TP3	2/35	10	Negativ^a^
Kantrowitz et al. 2019 [35]	89	F3	TP3	2/35	20	Negativ^a^
Lindenmayer et al. 2019 [43]	28	F3	TP3	2/35	20	Negativ
Valiengo et al. 2020 [71]	100	F3	TP3	2/35	20	Positiv

*TP3* temporoparietale Junktion (mittig zwischen T3 und P3), *FP2* rechts frontopolar, *F3/F4* linker bzw. rechter DLPFC (int. 10-20-EEG-System)

^a^Negativsymptomatik als sekundäres Outcomekriterium

### Weitere Effekte der tDCS

Zu den unmittelbaren klinischen Effekten scheint es auch noch weitere positive Effekte der tDCS zu geben. So wurden im Rahmen der tDCS-Forschung in den letzten Jahren mehrere Studien publiziert, die als Wirksamkeitskriterium nicht die primäre klinische Verbesserung, sondern eine Kognitionsverbesserung und eine Verbesserung des Cravings bei Tabakabhängigkeit unabhängig von der psychiatrischen Primärdiagnose untersuchten.

Angesichts dieser Ergebnisse wurde unter anderem das Craving, im Sinne eines Verlangens nach Zigaretten, in einer randomisierten, placebokontrollierten Studie mit 37 Schizophreniepatienten von Smith et al. [64] untersucht. Die Patienten wurden mit 2 mA tDCS (Anode F3, Kathode Fp2) in 5 Behandlungen präfrontal stimuliert. Hierbei zeigte sich keine Verbesserung psychiatrischer Symptome oder des Cravings, jedoch eine kognitive Verbesserung in der Verumgruppe im Vergleich zur Kontrollgruppe.

Außerdem gibt es Hinweise darauf, dass Tabakkonsum schizophrener Patienten unterschiedlichen Einfluss auf die Wirksamkeit der tDCS hat. Hierbei zeigte sich eine verbesserte tDCS-Wirkung auf akustische Halluzinationen bei Nichtrauchern [7].

Zudem konnten in mehreren Studien Kognitionsverbesserungen gezeigt werden. Nienow et al. [50] fanden eine signifikante Verbesserung der Kognition durch anodale tDCS bei 10 Patienten im Sinne besserer Ergebnisse eines Wort-und-Bild-2-back-Tests und der MATRICS Consensus Cognitive Battery (MCCB).

Vercammen et al. [73] fanden in diesem Zusammenhang keine Wirksamkeit der einmalig angewendeten tDCS im Sinne einer Kognitionsverbesserung. Bei einem Teil der 20 untersuchten Patienten fand sich jedoch eine Leistungssteigerung.

Eine Verbesserung des Arbeitsgedächtnisses von 18 Schizophreniepatienten zeigten Hoy et al. [28] in einer Crossover-Studie. Die Patienten erhielten präfrontale tDCS (Anode F3, Kathode Fp2) in 2 Verum- (1 und 2 mA) und einer Kontrollgruppe mit Scheinstimulation, mit dem Ergebnis einer Steigerung des Arbeitsgedächtnisses im n‑back-Test bis zu 40 min nach 2 mA Verum-tDCS im Vergleich zur Scheinstimulation und zu 1‑mA-tDCS.

Ebenso eine Verbesserung des Arbeitsgedächtnisses zeigten Orlov et al. [52]. Hierbei wurde eine doppelblinde, kontrollierte Studie an 49 Patienten durchgeführt, wobei sich signifikante Auswirkungen auf die Genauigkeit des Arbeitsgedächtnisses, jedoch keine Auswirkung auf die Lernfunktion nach tDCS-Behandlung ergaben.

Ribolsi et al. [56] untersuchten die Wirkung von tDCS auf 15 Patienten mit Schizophrenie und räumlichem Pseudoneglect. Hierbei zeigte eine anodale Stimulation des rechten parietalen Kortex (P4) eine Normalisierung im Line Bisection Test.

Die Effekte einer während des Schlafes durchgeführten Behandlung mit tDCS wurde durch Göder et al. [16] untersucht. Sie stimulierten 14 Schizophreniepatienten im Schlafstadium 2 mit langsam oszillierender tDCS (so-tDCS, 0,75 Hz, Anoden F3/F4, Kathode mastoidal) und fanden eine signifikante Verbesserung im Rey Auditory Verbal Learning Test. Eine weiter durchgeführte Scheinstimulation zeigte keinerlei Verbesserung im Vergleich.

In einer Open-label-Studie von Subramaniam et al. [68] mit 13 an Schizophrenie erkrankten Patienten und 10 Sitzungen mit 2 mA tDCS mit der Anode über F3 und der Kathode über TP3 wurden signifikante Verbesserungen im Eye Tracking Antisaccade Task berichtet. Außerdem verringerte sich der Schweregrad der akustischen Halluzinationen.

Interessant ist auch die Frage einer pharmakologischen Interaktion bei mit tDCS behandelten Schizophreniepatienten. Hierzu untersuchten Agarwal et al. [1] den Einfluss von Antipsychotika auf die Wirkung von tDCS an 36 Patienten. Vor allem weibliche Patientinnen, welche 10-mal mit 2 mA tDCS stimuliert wurden, zeigten eine geringere Verbesserung akustischer Halluzinationen bei gleichzeitiger Antipsychotikatherapie mit hoher D2-Rezeptor-Affinität als Patientinnen, die mit Antipsychotika mit niedriger D2-Rezeptor-Affinität behandelt wurden. Insgesamt ist jedoch über die Interaktion von tDCS mit antipsychotischer Medikation noch sehr wenig bekannt.

In den bisher durchgeführten Studien wurden von den behandelten Patienten keine spezifischen Nebenwirkungen im Rahmen der tDCS-Behandlung berichtet.

## tACS

Ein neues noch wenig erforschtes Hirnstimulationsverfahren stellt die tACS dar. Diese Methode beruht auf transkraniell appliziertem, sinusförmigem Wechselstrom in unterschiedlicher und individuell wählbarer Frequenz. Aufgrund der noch sehr wenigen vorhandenen Daten werden in dieser Übersicht auch Fallberichte und Fallserien dargestellt. Auf neurophysiologischer Ebene ist davon auszugehen, dass die sinusoidalen Schwingungen der tACS zu einer Modulation neuronaler Eigenfrequenzen führen, während die tDCS eine tonische Auslenkung des Ruhemembranpotenzials hervorruft. Die durch die tACS bedingte Modulation kortikaler Schwingungen scheint der Unterdrückung/Überlagerung der kortikalen Grundfrequenzen durch tDCS überlegen zu sein [69].

Erste Studien konnten zeigen, dass eine tACS mit 40 Hz (γ-Frequenz) zu einer Verbesserung der Gedächtnisleistung bei 10 gesunden Probanden führt [74], wohingegen eine Scheinstimulation in derselben Studie keine positiven Ergebnisse zeigte. Kallel et al. untersuchten die Wirksamkeit der tACS über dem dorsolateralen präfrontalen Kortex bei Patienten mit Schizophrenie, wobei sich hier eine Verringerung negativer Symptome zeigte [34].

In einer randomisierten, doppelblinden, kontrollierten Studie konnten Ahn et al. [2] zeigen, dass eine Stimulation mittels 10 Hz tACS pathologische kortikale α‑Oszillationen bei Patienten mit akustischen Halluzinationen verringert. Es wurden 22 Patienten mit Schizophrenie mit akustischen Halluzinationen untersucht, wobei diese 5 Tage lang jeweils 2‑mal täglich stimuliert wurden.

Kallel et al. [33] konnten in einer Fallserie an drei Clozapin-resistenten Patienten eine Abnahme negativer Symptome durch eine 4,5-Hz-Frequenz (θ) tACS über dem dorsolateralen präfrontalen Kortex hervorrufen. Hierbei erhielten die Patienten 20 Anwendungen in einer jeweiligen Dauer von 20 min.

In einer randomisierten, kontrollierten Studie mit 22 Patienten beschrieben Mellin et al. [43] eine nichtsignifikante Abnahme akustischer Halluzinationen im Rahmen der tACS. Hierbei wurden die Patienten randomisiert und je nach Studiengruppe 20 min mit einer Scheinstimulation, mit 10 Hz (2 mA) tACS oder 2 mA tDCS an 5 aufeinanderfolgenden Tagen behandelt.

Sreeraj et al. [66] zeigten mittels einer 5‑maligen täglichen 6 Hz tACS-Anwendung an einem Patienten mit Schizophrenie und kognitiven Defiziten eine Verbesserung des Arbeitsgedächtnisses und der Aufmerksamkeit. Interessant hierbei war, dass die positive Wirkung der tACS auch noch 50 Tage nach Beendigung der Behandlung nachweisbar war.

In einem Fallbericht von Sreeraj et al. [65] wurde eine Behandlung von γ‑tACS und θ‑tACS verglichen. Hierbei wurde die Stimulation jeweils in einer einzelnen Sitzung für 20 min appliziert, wobei sich bei der θ‑Frequenz die Kognition des Patienten verbesserte. Die γ‑tACS zeigte keine Veränderung.

Eine Studie von Hoy et al. [29], welche Scheinstimulation, tDCS und 40 Hz tACS verglich, zeigte an 11 Patienten mit Schizophrenie nach 20-minütiger Stimulation keine signifikante Wirkung der 40 Hz tACS bezüglich einer Verbesserung des Arbeitsgedächtnisses.

Haller et al. [19] konnten in einer Fallserie von drei Patienten, welche mit γ‑tACS 2‑mal täglich für jeweils 10 min über 2 Wochen stimuliert wurden, eine deutliche Verbesserung des PANSS, SANS und CDSS im Verlauf zeigen. Des Weiteren konnte eine Steigerung der kognitiven Funktionen beobachtet werden.

Haller et al. [20] beschreiben außerdem in einem weiteren Fallbericht über zwei Schizophreniepatienten, welche täglich 20 min lang mit γ‑tACS über 2 Wochen stimuliert wurden, eine ähnliche, wenn auch weniger deutliche Verbesserung der Symptome.

Im Rahmen der tACS-Behandlung wurden bisher keine stimulationsassoziierten Nebenwirkungen berichtet.

## tRNS

Als eine weitere Methode zur Behandlung der Negativsymptome bei Patienten mit Schizophrenie wurde in letzter Zeit die tRNS (transkranielle „random noise stimulation“) in kleineren Studien und Fallberichten untersucht. Anders als bei den bereits vorgestellten transkraniellen Stimulationsverfahren, bei denen die Patienten mit Gleich- bzw. Wechselstrom in fester Stromstärke bzw. Oszillationsfrequenz behandelt werden, erklärt sich die Funktion der tRNS über Applikation eines hochfrequent oszillierenden Stroms, dessen Oszillationsfrequenz, mit voreingestelltem Minimum- und Maximumwert, zufallsgesteuert wechselt [70]. Es wird angenommen, dass eine hochfrequente tRNS das Gehirn bzw. dessen Plastizität verändern kann und dieser Effekt teilweise sogar den der tDCS bzw. tACS übersteigt [72], weil die Neuronen aufgrund der schnellen Oszillationen nicht in der Lage sind, homöostatische Gegenregulationsprozesse gegen die Auslenkung des Ruhemembranpotenzials durchzuführen. Mulquiney et al. untersuchten hierzu 10 gesunde Probanden, die 3 Sitzungen lang jeweils über dem linken dorsolateralen präfrontalen Kortex (DLPFC) entweder 10 min anodale tDCS, tRNS oder eine Scheinstimulation erhielten [47]. Die Sitzungen waren mindestens eine Woche voneinander zeitlich getrennt. Hierbei konnten keine signifikanten Veränderungen auf die Arbeitsgedächtnisleistung der Probanden unter tRNS im Vergleich zu einer tDCS-Behandlung gezeigt werden. Palm et al. [53] zeigten in einem Fallbericht über einen 29-jährigen Patienten mit therapieresistenter Schizophrenie eine Symptomverbesserung nach 2 tRNS-Behandlungen zu je 20 min über 10 Tage. Hierbei lag die Anode über dem linken DLPFC und die Kathode rechts orbitofrontal bei Frequenzen zwischen 100 und 640 Hz. Es zeigte sich nach 20 Anwendungen eine Verbesserung der Negativsymptome des Patienten im Sinne einer Verringerung der getesteten Parameter wie PANSS, SANS und CDSS.

In einem weiteren Fallbericht von Haesebaert et al. wurde eine 26-jährige Patientin mit therapieresistenter Schizophrenie mit hochfrequenter tRNS (Frequenzen zwischen 100 und 640 Hz) für 20 min 2‑mal täglich an 5 aufeinanderfolgenden Tagen behandelt [18]. Hierbei konnte man nach 10 Sitzungen tRNS eine klinische Verbesserung sowie eine Verminderung der PANSS beobachten. Auch die Krankheitseinsicht der Patientin verbesserte sich. Die beschriebenen Veränderungen waren auch einen Monat nach der Intervention noch nachweisbar.

## Schlussbetrachtung

Zur Behandlung der Schizophrenie, speziell der Negativsymptomatik, stehen vielversprechende neue elektrische Hirnstimulationsverfahren zur Verfügung. Der wesentliche Vorteil dieser Therapieverfahren ist, neben ihrer guten Verträglichkeit und ihrer Kombinationsmöglichkeit mit Psychopharmakotherapie und Psychotherapie, die spezifische Interventionsmöglichkeit an pathophysiologisch relevanten Netzwerken. Die tDCS liefert als ergänzende Option zur Behandlung von Symptomen der Schizophrenie bislang einige, jedoch nicht durchgängig gute Ergebnisse bezüglich der Negativsymptomatik, ebenso wechselnde Ergebnisse in der Behandlung akustischer Halluzinationen bzw. weiterer Positivsymptomatik, weshalb die Datenlage insgesamt als heterogen zu bewerten ist. Es konnte neben einer Verringerung der Negativsymptomatik durch tDCS auch eine Verbesserung der Krankheitseinsicht gezeigt werden. Es wird vermutet, dass eine fehlende Krankheitseinsicht auf einer frontotemporalen Asymmetrie beruht, welche durch transkranielle Stimulationsverfahren beeinflusst wird [15]. Der erhoffte Effekt ist hierbei die Erhöhung der Motivation für die Teilnahme am alltäglichen Leben und an sozialer Interaktion und möglichst die Verhinderung einer frühzeitigen Erwerbsunfähigkeit mit allen daraus folgenden sozioökonomischen Nachteilen. Bislang wurde die tDCS als Zusatzbehandlung zur medikamentösen Therapie durchgeführt. Eine vergleichende Untersuchung zur Psychopharmakotherapie ist bislang nicht erfolgt. Vor einer Etablierung als alleinige Therapiemöglichkeit im Sinne einer Monotherapie müssen also weitere Studien folgen.

Die tACS als noch wenig untersuchte, aber möglicherweise vielversprechende Methode zur Behandlung der Schizophrenie bedarf noch weiterer Forschung. Weder über optimale Oszillationsfrequenz oder Elektrodenplatzierung noch über Stromstärke, Stimulationsdauer und Stimulationsanzahl gibt es bisher vergleichende Untersuchungen. In kleinen Fallserien konnte durch die tACS eine Verringerung der Negativsymptome und eine Verbesserung der kognitiven Funktionen bei Patienten mit Schizophrenie gezeigt werden. Hier sollten weitere, größer angelegte randomisierte, placebokontrollierte Studien folgen, um grundsätzlich die Wirkung und den Effekt bei psychischen Erkrankungen, und vor allem auf die Negativsymptomatik, beschreiben zu können.

Noch weniger Evidenz ist aktuell für die Verwendung der tRNS vorhanden. Zusammenfassend sei für tACS und tRNS bemerkt, dass die meisten Publikationen auf diesem Gebiet sich einer zu geringen Stichprobengröße an Patienten, meist als Einzelfallberichte, bedienen. Außerdem fehlen Studien zur Langzeitwirkung transkranieller Hirnstimulationsverfahren, selbst für das schon lang beforschte Verfahren tDCS.

Auch sollten Interaktionen der Medikation der Patienten sowie weitere mittlerweile bekannte Einflussfaktoren auf die Effektivität von NIBS-Verfahren (beispielsweise Nikotingebrauch, körperliche Aktivität, Händigkeit oder Unterschiede im Schlafverhalten) mit untersucht werden [30, 51, 67]. Inwieweit die Dauer der Stimulation, die Platzierung der Elektroden, die Anzahl der Behandlungen und das Intervall zwischen den Stimulationen von Bedeutung sind, muss zudem ebenfalls weiter beforscht werden, da auch diese Variablen als mögliche Einflussfaktoren auf die Effektivität der genannten Stimulationsverfahren diskutiert werden.

Vorteile der nichtinvasiven Behandlung sind vor allem eine nebenwirkungsarme Behandlung, eine leichte Handhabung der technischen Geräte, eine kostengünstige und flexible Anwendung sowie auch die prinzipiell mögliche Durchführbarkeit der Therapie beim Patienten zu Hause, z. B. in Form einer durch Video- oder Telefonkonferenz durch Fachpersonal unterstützten Fernbehandlung [55]. Damit reduziert sich die Dauer und Anzahl der Klinikaufenthalte und unterstützt die Selbstständigkeit und Therapieadhärenz des Patienten, was insbesondere bei Patienten mit Antriebsproblemen oder weiter Anreise von Bedeutung ist und dem politisch formulierten Wunsch nach „home treatment“ [26] nachkommt.

Zusammenfassend kann festgehalten werden, dass es auf dem Gebiet der nichtinvasiven transkraniellen Stimulationen neue vielversprechende und patientenfreundliche Methoden zur Behandlung der Schizophrenie bzw. einzelner Symptome gibt. Gleichzeitig besteht noch viel Forschungsbedarf zur klinischen Bedeutsamkeit und zu den Anwendungsparametern.

## Fazit für die Praxis

Neue nichtinvasive Hirnstimulationsverfahren könnten sich aufgrund ihrer einfachen Anwendbarkeit, der geringen Nebenwirkungen und der Kombinierbarkeit mit etablierten psychopharmakologischen und psychotherapeutischen Therapiestrategien zu einer relevanten Therapieoption für die Negativsymptomatik bei Schizophrenie entwickeln. Bisher liegen nur für die tDCS umfangreichere Daten vor, die für eine Wirksamkeit in dieser Indikation sprechen könnten. Für tACS und tRNS sind noch weitere Studien nötig, sie können gegenwärtig als experimentelle Verfahren angesehen werden.
